# The Scissors Effect in Action: The Fox-Flory Relationship between the Glass Transition Temperature of Crosslinked Poly(Methyl Methacrylate) and Mc in Nanophase Separated Poly(Methyl Methacrylate)-*l*-Polyisobutylene Conetworks

**DOI:** 10.3390/ma13214822

**Published:** 2020-10-28

**Authors:** Szabolcs Pásztor, Bálint Becsei, Györgyi Szarka, Yi Thomann, Ralf Thomann, Rolf Mühlhaupt, Béla Iván

**Affiliations:** 1 Polymer Chemistry Research Group, Institute of Materials and Environmental Chemistry, Research Centre for Natural Sciences, Hungarian Academy of Sciences, Magyar tudósok krt. 2, H-1117 Budapest, Hungary; modulus03@gmail.com (B.B.); szarka.gyorgyi@ttk.hu (G.S.); 2 Freiburg Center for Interactive Materials and Bioinspired Technologies (FIT), University of Freiburg, Georges-Köhler-Allee 105, D-79110 Freiburg, Germany; yi.thomann@fmf.uni-freiburg.de (Y.T.); ralf.thomann@fmf.uni-freiburg.de (R.T.); rolf.muelhaupt@makro.uni-freiburg.de (R.M.); 3 Freiburg Materials Research Center, University of Freiburg, Stefan-Meier-Str. 21, D-79104 Freiburg, Germany; 4 Institute for Macromolecular Chemistry, University of Freiburg, Stefan-Meier-Str. 31, D-79104 Freiburg, Germany

**Keywords:** polymer conetworks, poly(methyl methacrylate), polyisobutylene, DSC, AFM, glass transition temperature, Fox–Flory equation, scissors effect, bicontinuous nanophase separation

## Abstract

The glass transition temperature (*T_g_*) is one of the most important properties of polymeric materials. In order to reveal whether the *scissors effect*, i.e., the Fox–Flory relationship between *T_g_* and the average molecular weight between crosslinking points (*M_c_*), reported only in one case for polymer conetworks so far, is more generally effective or valid only for a single case, a series of poly(methyl methacrylate)-*l*-polyisobutylene (PMMA-*l*-PIB) conetworks was prepared and investigated. Two *T_g_s* were found for the conetworks by DSC. Fox–Flory type dependence between *T_g_* and *M_c_* of the PMMA component (*T_g_ = T_g,∞_* − *K/M_c_*) was observed. The *K* constants for the PMMA homopolymer and for the PMMA in the conetworks were the same in the margin of error. AFM images indicated disordered bicontinuous, mutually nanoconfined morphology with average domain sizes of 5–20 nm, but the correlation between *T_g_* and domain sizes was not found. These new results indicate that the macrocrosslinkers act like molecular scissors (*scissors effect*), and the *T_g_* of PMMA depend exclusively on the *M_c_* in the conetworks. Consequently, these findings mean that the *scissors effect* is presumably a general phenomenon in nanophase-separated polymer conetworks, and this finding could be utilized in designing, processing, and applications of these novel materials.

## 1. Introduction

Revealing the correlations between material properties and their structural characteristics is the key to designing, processing, and applications of materials. Among others, polymer conetworks (PCNs) belong to a recently emerged, widely investigated class of new macromolecular assemblies (see e.g., [1,2,3,4,5,6,7,8,9,10,11,12,13,14,15,16,17,18,19,20,21,22,23,24,25,26,27,28,29,30,31,32,33,34,35,36,37,38,39,40,41,42,43,44,45,46,47,48,49,50,51,52,53,54,55,56,57,58,59] and references therein). These novel crosslinked polymers, composed of chemically (covalently, ionically, or supramolecularly) bonded, mostly otherwise immiscible polymer chains, especially amphiphilic conetworks (APCNs) with hydrophilic and hydrophobic macromolecular components, have gained considerable attention in the last couple of years. This is mainly due to their unique bicontinuous (cocontinuous) nanophase-separated structure [12,17,33,34,35,36,37] and versatile application possibilities ranging from drug release matrices [43,44,45,46,47,48] to contact lenses [49,50], self-healing materials [4,58], membranes [24,51], sensors [20,52,53], catalyst supports [54,55], nanohybrids [56], and wound healing promoters [57], etc.

From the application point of view, one of the most important parameters of macromolecular materials is related to their glass transition temperatures (*T_g_s*). Below *T_g_*, polymers are solid, tough glassy, while above *T_g_*, they are visco-elastic, soft materials with rubbery properties in their crosslinked forms. However, in spite of the substantial importance of *T_g_s* of the components in the new PCNs, only relatively few reports have provided such data for conetworks so far [12,13,28,34,37,38,39], on the one hand. On the other hand, only one study by Fodor et al. [39] has been reported until now on attempts to find a correlation between *T_g_* of the components and structural parameters of poly(*N*-vinylimidazole)-*l*-poly(tetrahydrofuran) (PVIm-*l*-PTHF) conetworks, such as composition and average molecular weight of the PVIm chains between crosslinking points (*M_c_*). Surprisingly, it has been found that the *T_g_* of the crosslinked PVIm in the conetworks decreases with increasing crosslinking density, i.e., with decreasing *M_c_*. *T_g_s* of the crosslinked chains lower than that of the corresponding homopolymers have been found for other conetworks as well [13,35,36,43,44]. This is in sharp contrast to expectations according to which the *T_g_* of polymer networks crosslinked with low molecular weight crosslinkers increases even to very high *T_g_s* with increasing crosslinking density, i.e., with decreasing *M_c_* [60,61,62,63,64,65]. More astonishingly, the *T_g_* of PVIm in the conetworks versus the 1/*M_c_* plot has revealed that a Fox–Flory type correlation exists between the *T_g_* and *M_c_* of PVIm, independent of the molecular weight of the macromolecular crosslinker [39]. As known, according to the Fox–Flory equation [66], the *T_g_* of homopolymers is inversely proportional to their number average molecular weight: T_g_ = T_g,∞_ − K/M_n_, where *T_g,∞_* stands for the glass transition temperature of the polymer with infinite molecular weight, and K is a constant, as verified by experimental results for a variety of homopolymers [66,67,68,69,70,71,72,73]. Because this relationship has been found between the *T_g_* and *M_c_* of PVIm in the PVIm-*l*-PTHF conetworks, it has been concluded that the PTHF macromolecular crosslinker acts like atomic scissors by “cutting” the PVIm chains at the crosslinking points strictly from the point of view of the glass transition. As a consequence, the PVIm chains behave with respect to their glass transition like homopolymers between two crosslinking junctions with *M_n_* equal to their *M_c_*. Obviously, the question arises whether this “*scissors effect*” is valid exclusively only for the PVIm-*l*-PTHF conetworks, or this is a more general phenomenon for conetworks with immiscible polymer components.

Herein, we report on the synthesis of a series of poly(methyl methacrylate)-polyisobutylene (PMMA-*l*-PIB) conetworks by radical copolymerization of methyl methacrylate (MMA) with methacrylate-telechelic polyisobutylene (MA-PIB-MA) as a macromolecular crosslinker in order to investigate the effect of *M_c_* on the *T_g_* of PMMA in and the morphology of the resulting conetworks. For these systematic studies, the PMMA and MA-PIB-MA components have been selected partly on the basis of the large differences of their *T_g_s* (~100–120 °C for PMMA and around −70 °C for PIB) and, mainly, because of the matching structure of MMA and the methacrylate end group of the MA-PIB-MA bifunctional macromonomer. This affords to avoid the significant chemical differences and thus the large differences in the reactivity ratios of the comonomers, like in other conetwork syntheses, such as in the case of the copolymerization of N-vinylimidazole (VIm) and MA-PTHF-MA [38]. It has to be mentioned that PMMA-*l*-PIB conetworks by using three-arm star telechelic PIB were synthesized by Kennedy and Richard [74,75,76], aiming to obtain bone cement with improved impact resistance due to the biocompatibility of both PMMA and PIB. Although they determined the *T_g_s* of the components, attempts to investigate the effect of composition and/or *M_c_* on the *T_g_* of PMMA in such conetworks were not carried out. For this purpose, in the course of the present studies, we prepared PMMA-*l*-PIB PCNs with a broad range of compositions by using MA-PIB-MA macromonomers with several different molecular weights obtained by quasiliving carbocationic polymerization [77] and subsequent chain end derivatizations [34,77,78,79]. Then, systematic material characterizations were carried out by determining the gel fractions after the synthesis, the compositions, the *M_c_s*, the phase-separated morphologies, and the glass transition temperatures of the resulting PMMA-*l*-PIB conetworks, and the validity of the Fox–Flory equation between the *T_g_* and *M_c_* was examined on the basis of the obtained data in order to reveal whether the scissors effect exists in these crosslinked macromolecular materials.

## 2. Materials and Methods

### 2.1. Materials 

All chemicals used for the preparation of the tert-butyldicumyl chloride bifunctional initiator for quasiliving carbocationic polymerization of isobutylene and for the synthesis of methacrylate-telechelic polyisobutylene (MA-PIB-MA) and the poly(methyl methacrylate)-*l*-polyisobutylene (PMMA-*l*-PIB) conetworks were from commercial sources and purified by conventional purification techniques, as described earlier [80]. Methyl methacrylate (Sigma-Aldrich, St. Louis, MO, USA) was distilled under a vacuum before use.

### 2.2. Synthesis of Tert-Butyldicumyl Chloride and Telechelic Polyisobutylene Macromonomers

The three-step synthesis of the tert-butyldicumyl chloride bifunctional initiator was carried out, as described previously [78,79]. Methacrylate-telechelic polyisobutylenes (MA-PIB-MAs) were obtained by quasiliving carbocationic polymerization of isobutylene (Messer Hungarogáz Kft., Budapest, Hungary) initiated by the tert-butyldicumyl chloride/TiCl_4_ initiating system in the presence of N,N,N′,N′-tetramethylethylenediamine (TMEDA, 99.5%, Sigma-Aldrich, St. Louis, MO, USA) nucleophilic additive, followed by quantitative chain end derivatizations, as reported earlier [34,77,78,79]. Briefly, the polymerization of isobutylene was endquenched in situ by allyltrimethylsilane (ATMS, 97%, Honeywell Fluka, Charlotte, NC, USA) to obtain allyl-telechelic PIBs, and subsequent hydroboration/oxidation and esterification yielded the MA-PIB-MA macromonomers. The end products were purified by dissolving it in n-hexane (Molar Chemicals, Halásztelek, Hungary), passing through an alumina column, and precipitating in methanol (Molar Chemicals, Halásztelek, Hungary). Finally, the precipitated polymers were dried in a vacuum until constant weight at 30 °C. 

### 2.3. Synthesis of the PMMA-l-PIB Conetworks and PMMA Homopolymer

**Scheme 1:**
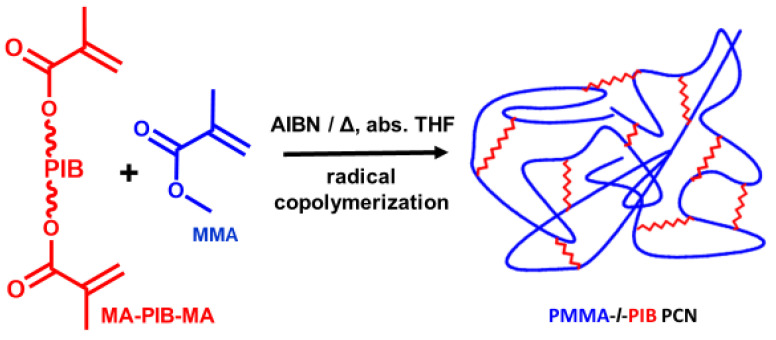
Synthesis of poly(methyl methacrylate)-*l*-polyisobutylene (PMMA-*l*-PIB) conetworks (the red wavy lines in the conetworks are the PIB cross-linkers).

The poly(methyl methacrylate)-*l*-polyisobutylene (PMMA-*l*-PIB) conetworks were synthesized, according to previously reported procedures [28,34,79,80], via the macromonomer method by free-radical copolymerization of methyl methacrylate (MMA) and the bifunctional macromonomer (MA-PIB-MA) with AIBN thermal radical initiator in tetrahydrofuran (THF) as common solvent at 65 °C for 72 h (Scheme 1). The reactions were carried out in three different feed compositions, i.e., 30/70, 50/50, and 70/30 wt% ratios, with MA-PIB-MA macromonomers of five different number average molecular weights. After the reaction, the conetworks were extracted with THF to remove the soluble fraction, that is, the non-reacted monomers and initiator, then dried under vacuum at 40 °C until constant weight. The ratios of the weights of the resulting extracted dry conetworks and the sum of the feed amounts of MMA and MA-PIB-MA gave the gel fractions. In one experiment, PMMA homopolymer (*M_n_* = 23,300 g/mol, *M_w_/M_n_* = 4.97) was also prepared under the same conditions as the conetworks.

### 2.4. Methods

^1^H NMR spectroscopy was used to obtain the chemical composition and the purity of the monomers and polymers and the functionality of the telechelic macromonomers used in this work. ^1^H NMR spectra were obtained on a Bruker Avance-I spectrometer (Bruker, Bremen, Germany) operating at a ^1^H frequency of 250 MHz. Samples were dissolved in CDCl_3_. TMS (tetramethylsilane) at 0 ppm and CDCl_3_ at 7.28 ppm were used as internal references. 

The molecular weight distributions (MWD) and average molecular weights of the MA-PIB-MA macromonomers, precursors, and the PMMA homopolymer were measured by gel permeation chromatography (GPC). The GPC was composed of a Waters 515 HPLC (High Performance Liquid Chromatography) pump (Waters, Milford, MA, USA), Waters Styragel column (Waters, Milford, MA, USA) set with three columns (HR1, HR2, HR4), and it was equipped with an RI detector. THF was used as mobile phase with a flow rate of 1 mL/min. The average molar masses, as well as the polydispersity (*M_w_/M_n_*), were determined and calculated by the use of a calibration made with narrow MWD polystyrene standards in the molecular weight range of 104 (styrene) to 3 × 10^6^ Da. 

Elemental analysis carried out with Heraeus CHN-O-RAPID equipment (Heraeus, Hanau, Hessen, Germany) was used to determine the composition of the conetwork samples. 

Atomic force microscopy (AFM) measurements of the PMMA-*l*-PIB conetwork samples were carried out by height and phase mode settings with a MultiMode scanning probe microscope having Nanoscope IIIa controller (Digital Instruments) on sample surfaces obtained by cryosectioning at −120 °C with a Leica EMFCS microtome (Leica, Wetzlar, Hessen, Germany) equipped with a Diatome diamond knife.

The analyses of the homopolymers and the PMMA-*l*-PIB conetworks by differential scanning calorimetry (DSC) were performed under a nitrogen atmosphere with a Mettler Toledo TC15 equipment (Mettler Toledo, Leicester, UK) in the temperature range of −120 °C–180 °C by heating and cooling rates of 10 °C/min. The inflection points of the transition ranges of the DSC traces of the second heating run were used for the determination of the glass transition temperatures.

## 3. Results and Discussion

Methacrylate-telechelic polyisobutylenes (MA-PIB-MA) with narrow molecular weight distributions were obtained by quasiliving carbocationic polymerization of isobutylene, followed by in-situ formation of allyl chain ends via end-quenching with ATMS [76,77]. Subsequent quantitative hydroboration/oxidation and esterification with methacryloyl chloride led to hydroxyl and methacrylate-telechelic polyisobutylenes (PIBs), respectively [28,78,79,80]. The resulting MA-PIB-MA macromonomers were characterized in terms of their molecular weight distributions (MWD), average molecular weights, average end functionality, and glass transition temperatures, as shown in Table 1 (see Figures S1–S11 for the MWDs and ^1^H NMR spectra in the Supplementary Materials). These data indicated that a series of MA-PIB-MA macromonomers with *M_n_* in the range of 2300–13,300 g/mol and functionality of two was obtained. These samples were denoted with their *M_n_* values (kDa) obtained by ^1^H NMR measurements.

As depicted in Scheme 1, a series of PMMA-*l*-PIB conetworks, listed in Table 2, was prepared by radical copolymerization of MMA with the MA-PIB-MAs with three different MA-PIB-MA/MMA feed ratios, i.e., 30/70, 50/50, and 70/30 wt%. In the sample codes of the resulting PMMA-*l*-PIB conetworks, the first numbers represented the *M_n_* values divided by 1000, and the second ones were the weight fractions of the MA-PIB-MA macrocrosslinkers in the feed. 

As shown in Table 2, the copolymerizations of MMA with MA-PIB-MA resulted in high gel fractions, between 90% and 100%, with the exception of the PMMA-*l*-PIB-13.30-30 sample having 86% gel fraction. This could be attributed to the relatively high *M_n_* and, thus, to the low concentration of the macrocrosslinker in this case. The nearly quantitative formation of the insoluble crosslinked polymers indicated high crosslinking efficiency in all the investigated conetwork syntheses. The successful conetwork syntheses were also corroborated by the close to feed ratio compositions of extracted conetworks, as obtained by elemental analyses (Table 2). As found earlier in the course of similar conetwork syntheses, the methacrylate-telechelic macromonomer was completely consumed during such processes [81], and thus the average molecular weight between two crosslinking points of the PMMA chains (*M_c_*) could be obtained by the following formula:

*M_c_ = 0.5·W_PMMA_·M_n,PIB_/W_PIB_* (1)

where *W_PMMA_*, *W_PIB_,* and *M_n,PIB_* denote the weight fractions of PMMA and PIB and the *M_n_* of the PIB crosslinker, respectively. As shown in Table 2, PMMA-*l*-PIB conetworks with *M_c_s*, covering more than one order of magnitude in the range of 700−12,000 g/mol, were obtained with the applied MA-PIB-MA macromonomers and feed ratios.

**Figure 1:**
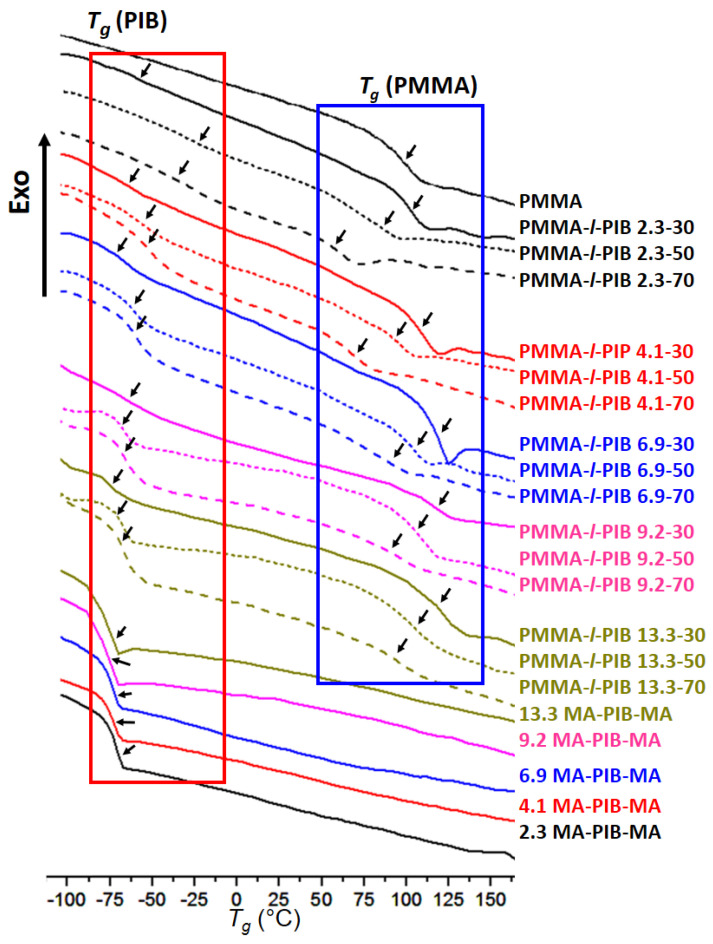
Glass transition temperatures (*T_g_*) of the poly(methyl methacrylate)-*l*-polyisobutylene (PMMA-*l*-PIB) conetworks as a function of composition (the lines represent the *T_g_* of the methacrylate-telechelic polyisobutylenes (MA-PIB-MA) macromolecular cross-linkers and the poly(methyl methacrylate) (PMMA) homopolymer, and the inflection points as *T_g_* values are indicated by arrows).

The differential scanning calorimetry (DSC) curves of the PMMA-*l*-PIB conetworks and the PMMA and MA-PIB-MA homopolymers are displayed in Figure 1. As shown in this figure and by the data in Table 2, the PMMA prepared by us had a *T_g_* at 99 °C, while the *T_g_s* of the MA-PIB-MA telechelic macromonomers fell in the region from −72 °C to −65 °C. The DSC scans of the PMMA-*l*-PIB conetworks showed that all the conetworks, regardless of composition and molecular weight (MW) of the MA-PIB-MA crosslinkers, had two distinct glass transition regions. One of the *T_g_* regions appeared at low temperatures close to that of the MA-PIB-MA homopolymers, while the other one could be found at higher temperatures close to but with well observable differences from the *T_g_* of the PMMA homopolymer. The existence of the two *T_g_s* in the PMMA-*l*-PIB conetworks definitely indicated that these transparent materials possessed a phase-separated structure, i.e., separate PMMA and PIB domains existed in these crosslinked polymers. According to the data in Table 2, the *T_g_s* of the PMMA components in the conetworks covered a broad range from 57 °C to 119 °C. 

**Figure 2:**
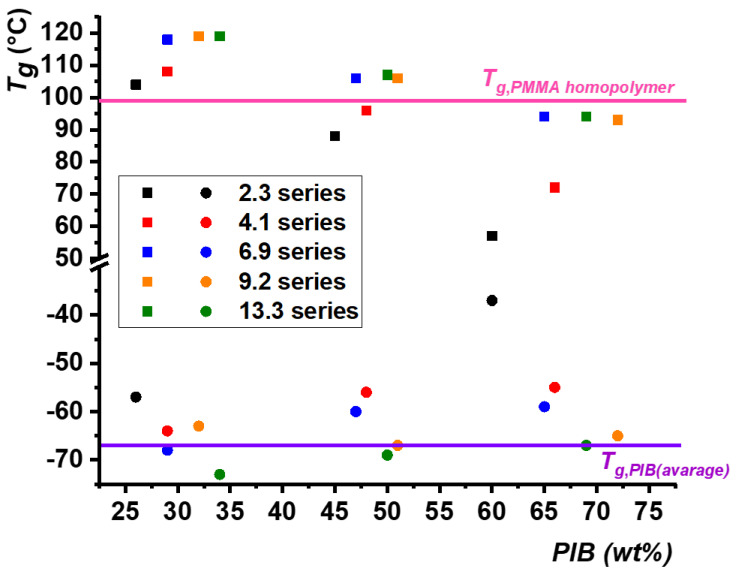
Glass transition temperatures (*T_g_*) of the PMMA-*l*-PIB conetworks as a function of composition (the lines represent the *T_g_* of the MA-PIB-MA macromolecular cross-linkers and the PMMA homopolymer).

As displayed in Figure 2, there was a monotonous decrease of the *T_g_* of PMMA with the increase of the PIB content within each conetwork series with a given *M_n_* of the MA-PIB-MA macromolecular crosslinker. It can also be seen in this figure that the higher the *M_n_* of the MA-PIB-MA macromonomers, the higher the *T_g_* of the PMMA component in the conetworks at the same composition. As shown in Figure 2, the *T_g_s* of the PMMA in the conetworks at relatively low PIB contents appeared at higher than that of the PMMA homopolymer. Similar results were reported by Kennedy and Richard [74], who applied only 5–30 wt% trifunctional methacrylate-telechelic PIB as a crosslinker and found somewhat higher *T_g_* of PMMA in their conetworks than that of the homopolymer in some cases. This effect might be due to the expected increase of *T_g_* of crosslinked polymers, as reported in the literature [60,61,62,63,64,65]. However, in sharp contrast to these expectations, the *T_g_* of the PMMA component decreased significantly, even below the *T_g_* of the PMMA homopolymer, with the increase of the weight fraction of the MA-PIB-MA macrocrosslinker in the conetworks, i.e., with the increasing crosslinking density, and thus with the decreasing M_c_ of the PMMA chains.

**Figure 3:**
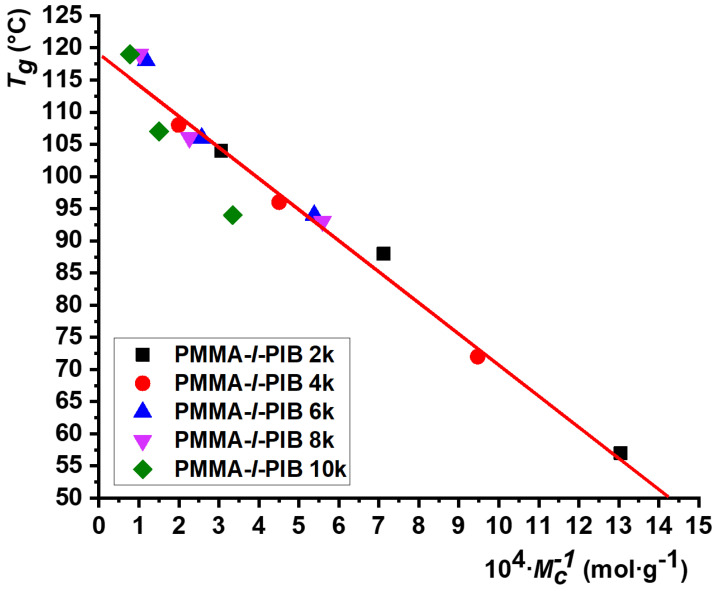
The Fox–Flory plot of the glass transition temperatures: *T_g_s* of the PMMA component as a function of 1/*M_c_* in the PMMA-*l*-PIB conetworks.

Plotting the *T_g_* values of the PMMA component in the PMMA-*l*-PIB conetworks as a function of 1/*M_c_* in Figure 3 clearly indicated that the *T_g_* of PMMA linearly decreased with increasing 1/*M_c_*, independent of the *M_n_*, i.e., the chain length, of the PIB macrocrosslinkers. This finding was similar to what Fodor et al. [39] reported for the poly(*N*-vinylimidazole)-*l*-poly(tetrahydrofuran) (PVIm-*l*-PTHF) conetworks; that is, the linear relationship between *T_g_* and 1/*M_c_* followed the Fox–Flory equation [66] for the crosslinked PMMA in the conetworks by substituting *M_n_* with *M_c_*, i.e., with the average molecular weight between two crosslinking points:

*T_g_ = T_g,∞_* − *K/M_c_* (2)

The slope of the straight line in Figure 3 was 4.83 × 10^4^ °C·g/mol. Unexpectedly, this was nearly identical with the K constant of 4.67 × 10^4^ °C·g/mol reported for the PMMA homopolymer with *M_n_* range of 300–14,000 g/mol [67]. Taking into account that the *M_c_s* in the PMMA-*l*-PIB conetworks fall in the same region (~700–13,000 g/mol), this result meant that the crosslinked PMMA chains between two branching points in the conetworks behaved like the PMMA homopolymers from the point of view of the glass transition. This finding indicated that the PIB macrocrosslinkers acted like scissors for the PMMA chains in the PMMA-*l*-PIB conetworks, on the one hand. On the other hand, in addition to the similar results with the PVIm-*l*-PTHF conetworks, this was the second example of the validity of the *scissors effect* in polymer conetworks, indicating that this phenomenon can be more general in the case of PCNs composed of immiscible polymer chains than one single example for the PVIm-*l*-PTHF conetworks [39].

**Figure 4:**
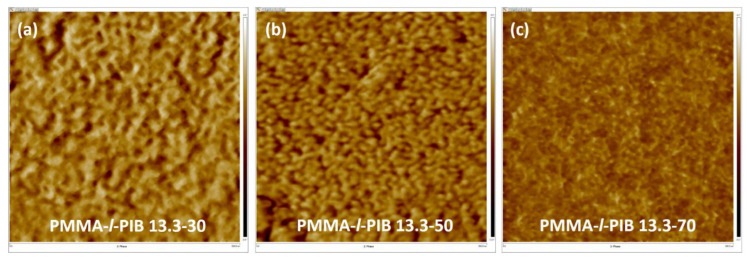
Atomic force microscopy (AFM) phase mode images of the cross-section of the PMMA-*l*-PIB-13.3 conetwork series (bright phases: PMMA, dark phases: PIB; picture dimensions: 500 nm × 500 nm); (**a**) PMMA-*l*-PIB 13.3-30, (**b**) PMMA-*l*-PIB 13.3-50, and (**c**) PMMA-*l*-PIB 13.3-70).

In order to verify the phase-separated structure of the PMMA-*l*-PIB conetworks, AFM measurements were carried out. Figure 4 shows a series of typical AFM images of the PMMA-*l*-PIB-13.3 conetwork series. In these AFM images, the softer PIB phases appeared dark, while the harder PMMA domains were bright. In agreement with the DSC results, these images clearly showed the existence of phase separation in these conetworks, on the one hand. On the other hand, it can also be seen that disordered bicontinuous (cocontinuos), i.e., mutually nanoconfined, phase structure was present in the conetworks with 34 wt% and 50 wt%, while individual small PMMA domains also appeared in the conetworks with higher (69 wt%) PIB contents. The bicontinuous nanophasic morphology of the PMMA-*l*-PIB conetworks in a broad composition range (~30–50 wt% PIB) was in good agreement with previous results for other conetworks [12,36,37].

The AFM images also revealed that the average domain sizes, determined by image analysis, decreased from about 20 nm to 7 nm on average with the increase of the PIB content from 34 wt% to 69 wt%, i.e., with the increase of the crosslinking density. However, it has to be noted that an acceptable correlation between the *T_g_s* of the PMMA and its average domain sizes was not found. This also indicated that the *T_g_* of the PMMA chains in the PMMA-*l*-PIB conetworks was determined by the *scissors effect* of the macromolecular crosslinker, that is, exclusively by one structural parameter, the *M_c_* of PMMA, in these unique materials, according to the Fox–Flory type correlation between *T_g_* and *M_c_*.

## 4. Conclusions

On the basis of the previous unprecedented findings on the *scissors effect* in PVIm-*l*-PTHF conetworks [39], i.e., on the Fox–Flory type relationship between *T_g_* and *M_c_*, a series of PMMA-*l*-PIB conetworks was synthesized in a broad range of composition and MW of the MA-PIB-MA macrocrosslinker and was investigated in order to reveal whether this is a more general phenomenon in these emerging novel materials or occurs only in a single case, i.e., in PVIm-*l*-PTHF. Two *T_g_s* in the region of the homopolymers were observed by DSC measurements for the PMMA-*l*-PIB conetworks, indicating phase separation of the components in these materials. The *T_g_s* of PMMA were in the 57–119 °C region and decreased with increasing PIB content, i.e., with decreasing *M_c_* for all the conetwork series. This was in sharp contrast to PMMA networks having significantly increased *T_g_s* by crosslinking with low MW crosslinkers, on the one hand. On the other hand, Fox–Flory relationship was found between the *T_g_* of the crosslinked PMMA in the conetworks and its *M_c_*, i.e., *T_g_* = *T_g,∞_* − K/M_c_. Consequently, it can be concluded that the *scissors effect* by the macromolecular crosslinker, strictly from the point of view of glass transition, was operative in the PMMA-*l*-PIB conetworks. Surprisingly, the K constant of the PMMA in the polymer conetworks (4.83 × 10^4^ °C·g/mol), obtained by plotting *T_g_s* according to the Fox–Flory equation, was nearly the same within experimental error as that reported for the PMMA homopolymer (4.67 × 10^4^ °C·g/mol) [67]. This meant that the PMMA chains between crosslinking points from the point of view of glass transition behaved as the PMMA homopolymers with the same MW as the *M_c_* in the conetworks. AFM measurements showed that the PMMA-*l*-PIB conetworks possessed nanophase-separated, mutually nanoconfined bicontinuous (cocontinuous) disordered morphology with average domain sizes of ~7–20 nm. However, the correlation between *T_g_* and the domain sizes was not found, indicating that exclusively, the *M_c_* was the only structural parameter, which determined the *T_g_* of the crosslinked PMMA in the PMMA-*l*-PIB conetworks. 

One may consider applying the so-called “self-concentration” approach, developed by Lodge and McLeish [82], for the interpretation of the glass transition temperatures of miscible blends, for the explanation of the observed tendency of the *T_g_s* in the conetworks. However, there are two major differences that should be taken into account. One is obviously the structural aspect, i.e., the conetworks are crosslinked polymers with distinct crosslinking points and not blends with freestanding polymer chains, while the other relates to the fact that PMMA and PIB are immiscible polymers. It should also be noted that according to the “self-concentration” in miscible blends, the *T_g_* of the polymer component with the lower *T_g_*, PIB, in this case, should increase with decreasing PIB content. As shown in Figure 1 and Figure 2, this is evidently not the case with conetworks. The same trend was obtained for the polymer with the lower *T_g_*, PTHF, in the PVIm-*l*-PTHF conetworks as well [37]. These unprecedented aspects with other unique features of conetworks of immiscible polymers, in accordance with the claims in a recent overview on polymer conetworks by Patrickios and Matyjaszewski [83], require future research to reveal several, still not fully explored fundamental and structural details of the structure-property relationships of polymer conetworks, including the observed Fox–Flory relationship between the *T_g_* and *M_c_* in these crosslinked polymer assemblies as well. 

In sum, the results presented in this study confirm that the *scissors effect* by the macromolecular crosslinkers for the crosslinked chains in polymer conetworks exists not only for PVIm-*l*-PTHF but in the case of PMMA-*l*-PIB conetworks as well. Thus, this new example of the *scissors effect* in conetworks indicates that this is presumably a general phenomenon in polymer conetworks. As a consequence, because *T_g_* is one of the most important parameters of macromolecular materials, these results should be considered and utilized in the design and applications of this novel class of materials.

## Figures and Tables

**Table 1:** The number average molecular weights (*M_n_*) by ^1^H NMR spectroscopy and GPC, the polydispersities (*M_w_/M_n_*), and the glass transition temperatures (*T_g_*) of methacrylate-telechelic polyisobutylenes (MA-PIB-MAs).

Sample	*M_n_* (^1^H NMR) (g/mol)	*M_n_* (GPC) (g/mol)	*M_w_/M_n_*	*T_g_* (°C)
MA-PIB-MA 2.3	2300	2600	1.06	−65
MA-PIB-MA 4.1	4100	4500	1.15	−66
MA-PIB-MA 6.9	6900	6800	1.12	−66
MA-PIB-MA 9.2	9200	9100	1.13	−72
MA-PIB-MA 13.3	13,300	11,900	1.07	−70

**Table 2:** The gel fractions, compositions (PIB/PMMA wt% ratios), the average molecular weights between crosslinks (*M_c_*), and the glass transition temperatures (*T_g_*) of PMMA-*l*-PIB conetworks.

Sample	Gel Fraction (%)	PIB/PMMA Ratio (wt%)	*M_c,PMMA_* (g/mol)	*T_g_* (°C) (PMMA)	*T_g_* (°C) (PIB)
PMMA-*l*-PIB 2.3-30	96	26/74	3270	104	−57
PMMA-*l*-PIB 2.3-50	97	45/55	1400	88	−23
PMMA-*l*-PIB 2.3-70	96	60/40	770	57	−37
PMMA-*l*-PIB 4.1-30	91	29/71	5020	108	−64
PMMA-*l*-PIB 4.1-50	95	48/52	2220	96	−56
PMMA-*l*-PIB 4.1-70	95	66/34	1060	72	−55
PMMA-*l*-PIB 6.9-30	95	29/71	8450	118	−68
PMMA-*l*-PIB 6.9-50	96	47/53	3890	106	−60
PMMA-*l*-PIB 6.9-70	96	65/35	1860	94	−59
PMMA-*l*-PIB 9.2-30	90	32/68	9770	119	−63
PMMA-*l*-PIB 9.2-50	100	51/49	4420	106	−67
PMMA-*l*-PIB 9.2-70	93	72/28	1790	93	−65
PMMA-*l*-PIB 13.3-30	86	34/66	12,910	119	−73
PMMA-*l*-PIB 13.3-50	93	50/50	6650	107	−69
PMMA-*l*-PIB 13.3-70	92	69/31	2990	94	−67

## Supplementary Materials

The following are available online at https://www.mdpi.com/1996-1944/13/21/4822/s1, Figure S1: The molecular weight distribution of the MA-PIB-MA2.3 methacrylate-telechelic polyisobutylene obtained by GPC measurement (*M_n_* = 2600 g/mol, *M_w_/M_n_* = 1.06), Figure S2: The molecular weight distribution of the MA-PIB-MA4.1 methacrylate-telechelic polyisobutylene obtained by GPC measurement (*M_n_* = 4500 g/mol, *M_w_/M_n_* = 1.15), Figure S3: The molecular weight distribution of the MA-PIB-MA6.9 methacrylate-telechelic polyisobutylene obtained by GPC measurement (*M_n_* = 6800 g/mol, *M_w_/M_n_* = 1.12), Figure S4: The molecular weight distribution of the MA-PIB-MA9.2 methacrylate-telechelic polyisobutylene obtained by GPC measurement (*M_n_* = 9100 g/mol, *M_w_/M_n_* = 1.13), Figure S5: The molecular weight distribution of the MA-PIB-MA13.3 methacrylate-telechelic polyisobutylene obtained by GPC measurement (*M_n_* = 11,900 g/mol, *M_w_/M_n_* = 1.07), Figure S6: The molecular weight distribution of the PMMA obtained by GPC measurement (*M_n_* = 23,300, *M_w_/M_n_* = 4.97), Figure S7: ^1^H NMR spectrum of the MA-PIB-MA2.3 sample, Figure S8: ^1^H NMR spectrum of the MA-PIB-MA4.1 sample, Figure S9: ^1^H NMR spectrum of the MA-PIB-MA6.9 sample, Figure S10: ^1^H NMR spectrum of the MA-PIB-MA9.2 sample, Figure S11: ^1^H NMR spectrum of the MA-PIB-MA13.3 sample.
