# Supplementary material for: The Scissors Effect in Action: The Fox-Flory Relationship between the Glass Transition Temperature of Crosslinked Poly(Methyl Methacrylate) and Mc in Nanophase Separated Poly(Methyl Methacrylate)-l-Polyisobutylene Conetworks
Source: Materials (Basel). 2020 Oct 28;13(21):4822. doi: 10.3390/ma13214822 (PMC7663353; doi:10.3390/ma13214822)
Supplement: Supplementary file 1 [file materials-13-04822-s001.pdf]

materials

MDPI

Supporting Materials

The Scissors Effect in Action: The Fox-Flory Relationship Between the Glass Transition Temperature of Crosslinked Poly(Methyl Methacrylate) and Mc in Nanophase Separated Poly(Methyl Methacrylate)-*l*-Polyisobutylene Conetworks

Szabolcs Pásztor [1,*], Bálint Becsei [1], Györgyi Szarka [1], Yi Thomann [2], Ralf Thomann [2,3], Rolf Mühlhaupt [2,3,4] and Béla Iván [1,*]

[1] Polymer Chemistry Research Group, Institute of Materials and Environmental Chemistry, Research Centre for Natural Sciences, Hungarian Academy of Sciences, Magyar tudósok krt. 2, H-1117 Budapest, Hungary; modulus03@gmail.com (B.B.); szarka.gyorgyi@ttk.hu (G.S.)

[2] Freiburg Center for Interactive Materials and Bioinspired Technologies (FIT), University of Freiburg, Georges-Köhler-Allee 105, D-79110 Freiburg, Germany; yi.thomann@fmf.uni-freiburg.de (Y.T.); ralf.thomann@fmf.uni-freiburg.de (R.T.); rolf.muelhaupt@makro.uni-freiburg.de (R.M.)

[3] Freiburg Materials Research Center, University of Freiburg, Stefan-Meier-Str. 21, D-79104 Freiburg, Germany

[4] Institute for Macromolecular Chemistry, University of Freiburg, Stefan-Meier-Str. 31, D-79104 Freiburg, Germany

*Correspondence: pasztor.szabolcs@ttk.hu (S.P.), ivan.bela@ttk.hu (B.I.)

Received: 4 October 2020; Accepted: 24 October 2020; Published: 28 October 2020

Content

Molecular weight distribution curves of MA–PIB–MA and PMMA samples (Figures S1–S6)

^{1}H NMR spectra of MA–PIB–MA samples (Figures S7–S11)

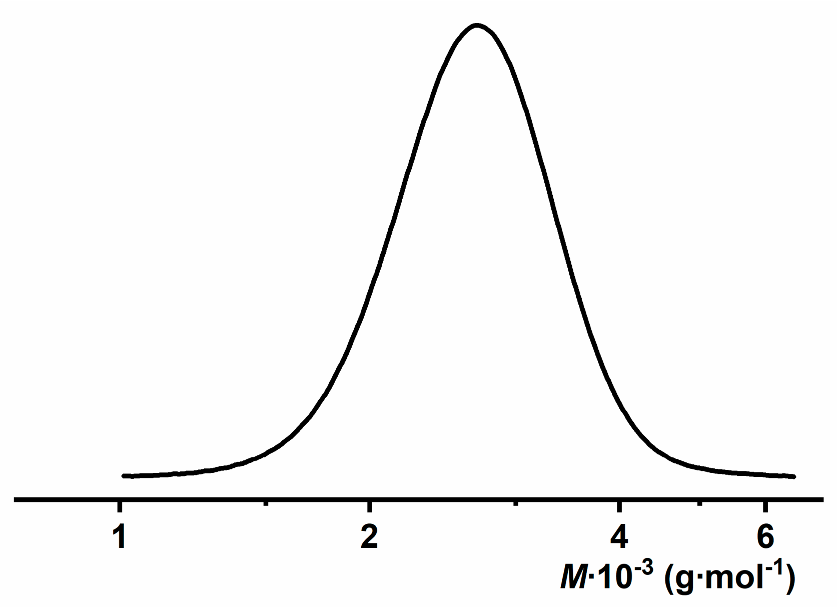

Figure S1. The molecular weight distribution of the MA–PIB–MA2.3 methacrylate–telechelic polyisobutylene in logarithmic scale obtained by GPC measurement (M_n = 2600 g/mol, M_w/M_n = 1.06).

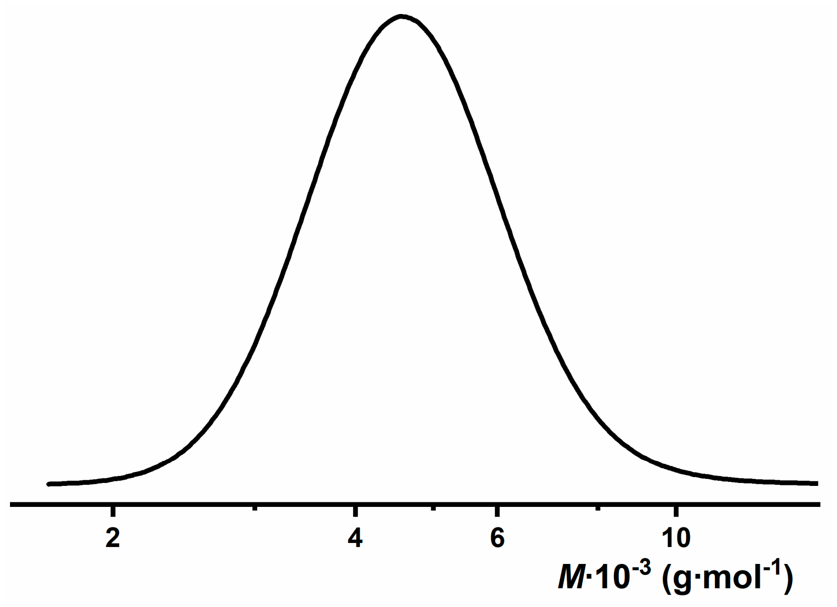

Figure S2. The molecular weight distribution of the MA–PIB–MA4.1 methacrylate–telechelic polyisobutylene in logarithmic scale obtained by GPC measurement (M_n = 4500 g/mol, M_w/M_n = 1.15).

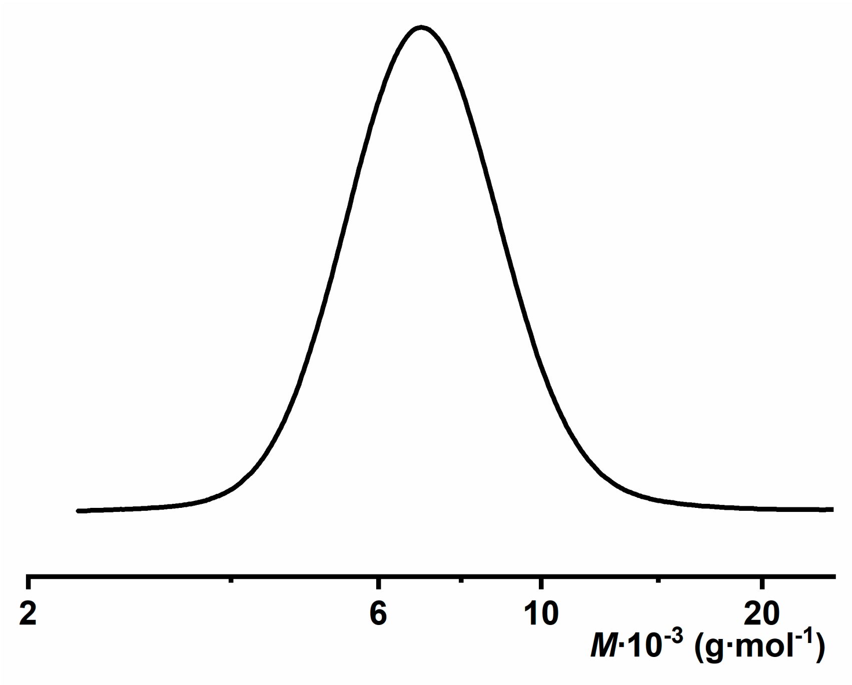

Figure S3. The molecular weight distribution of the MA–PIB–MA6.9 methacrylate–telechelic polyisobutylene in logarithmic scale obtained by GPC measurement (M_n = 6800 g/mol, M_w/M_n = 1.12).

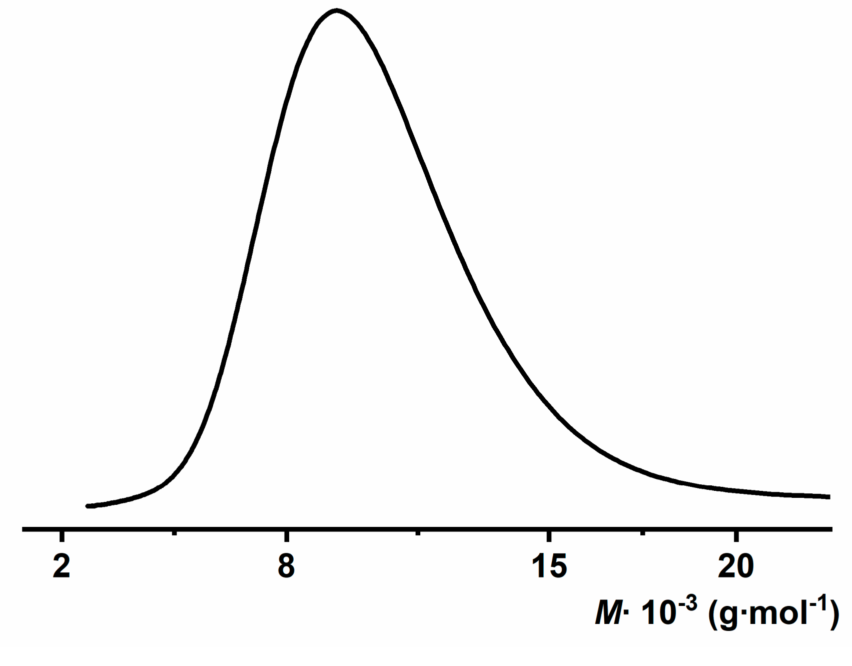

Figure S4. The molecular weight distribution of the MA–PIB–MA9.2 methacrylate–telechelic polyisobutylene in logarithmic scale obtained by GPC measurement (M_n = 9100 g/mol, M_w/M_n = 1.13)

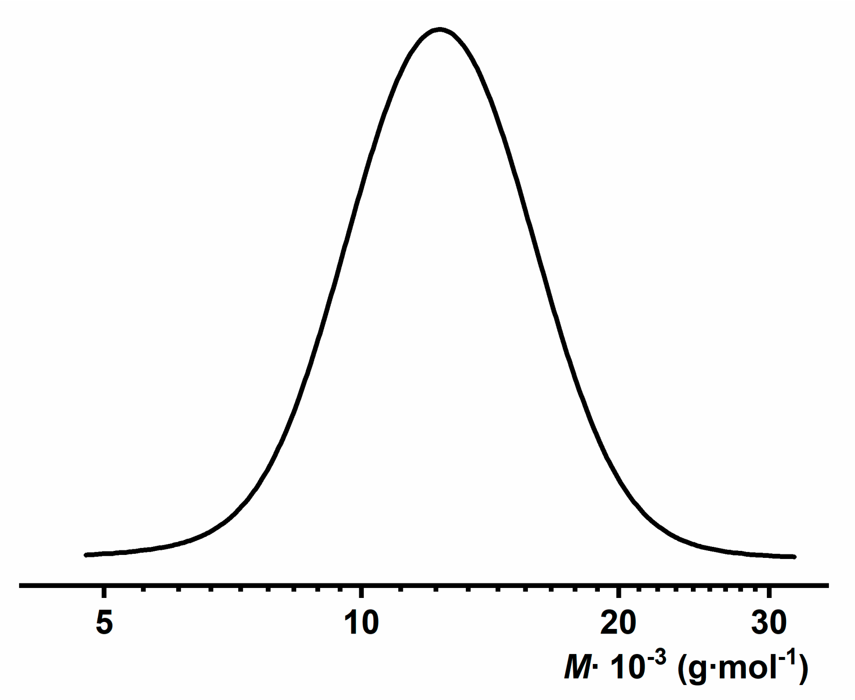

Figure S5. The molecular weight distribution of the MA–PIB–MA13.3 methacrylate–telechelic polyisobutylene in logarithmic scale obtained by GPC measurement (M_n = 11,900 g/mol, M_w/M_n = 1.07).

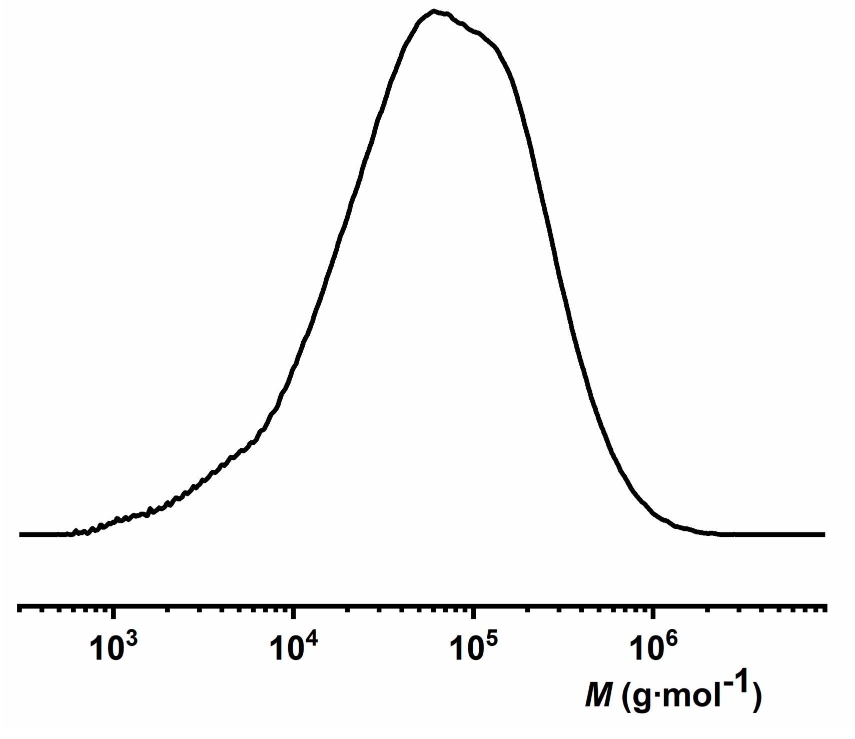

Figure S6. The molecular weight distribution of the PMMA in logarithmic scale obtained by GPC measurement (M_n = 23,300, M_w/M_n = 4.97).

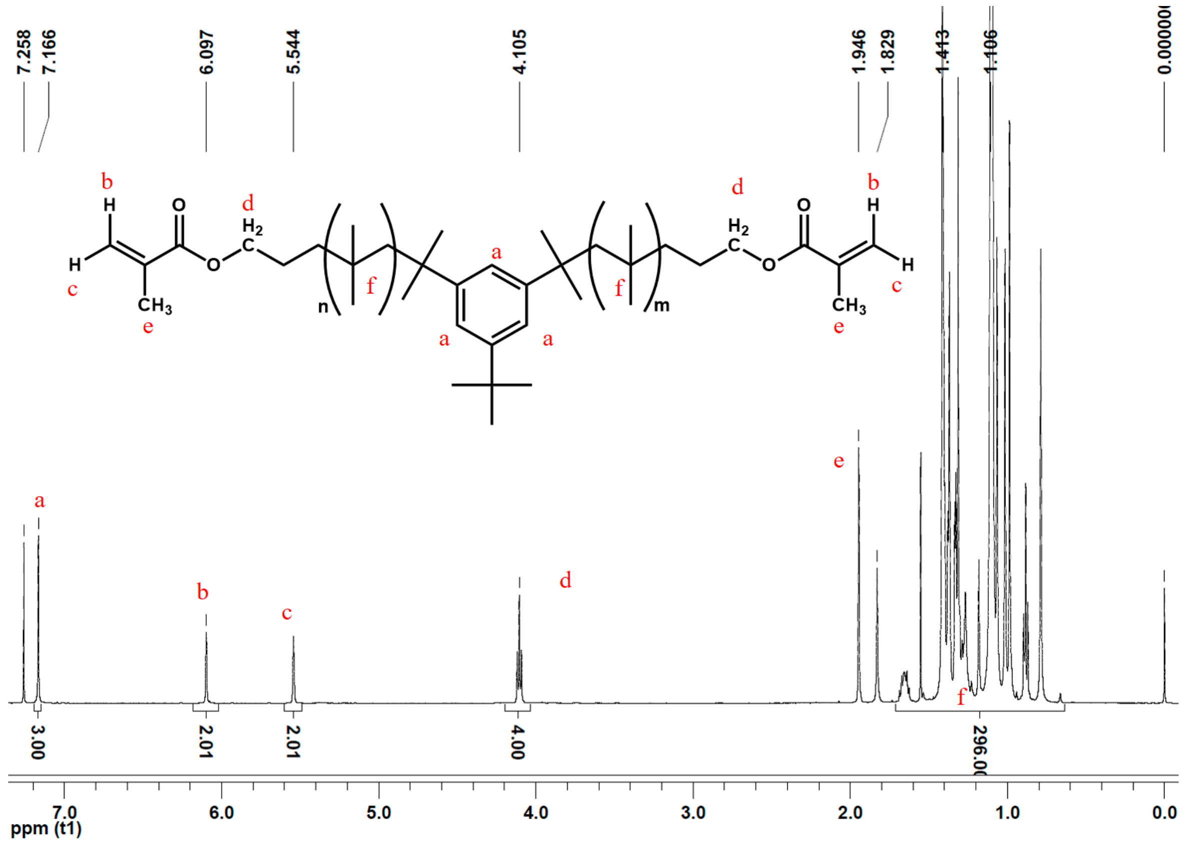

Figure S7. ^{1}H NMR spectrum of the MA–PIB–MA2.3 sample**.**

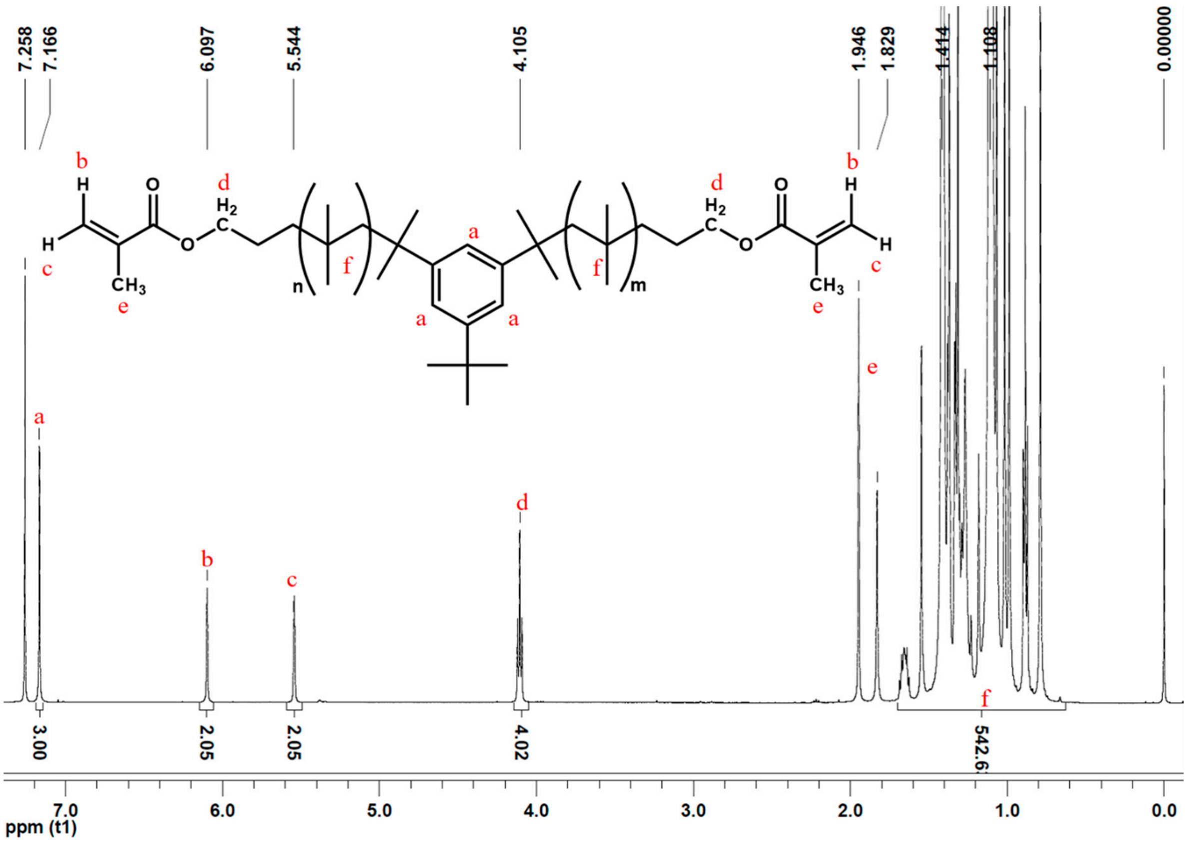

Figure S8. ^{1}H NMR spectrum of the MA–PIB–MA4.1 sample.

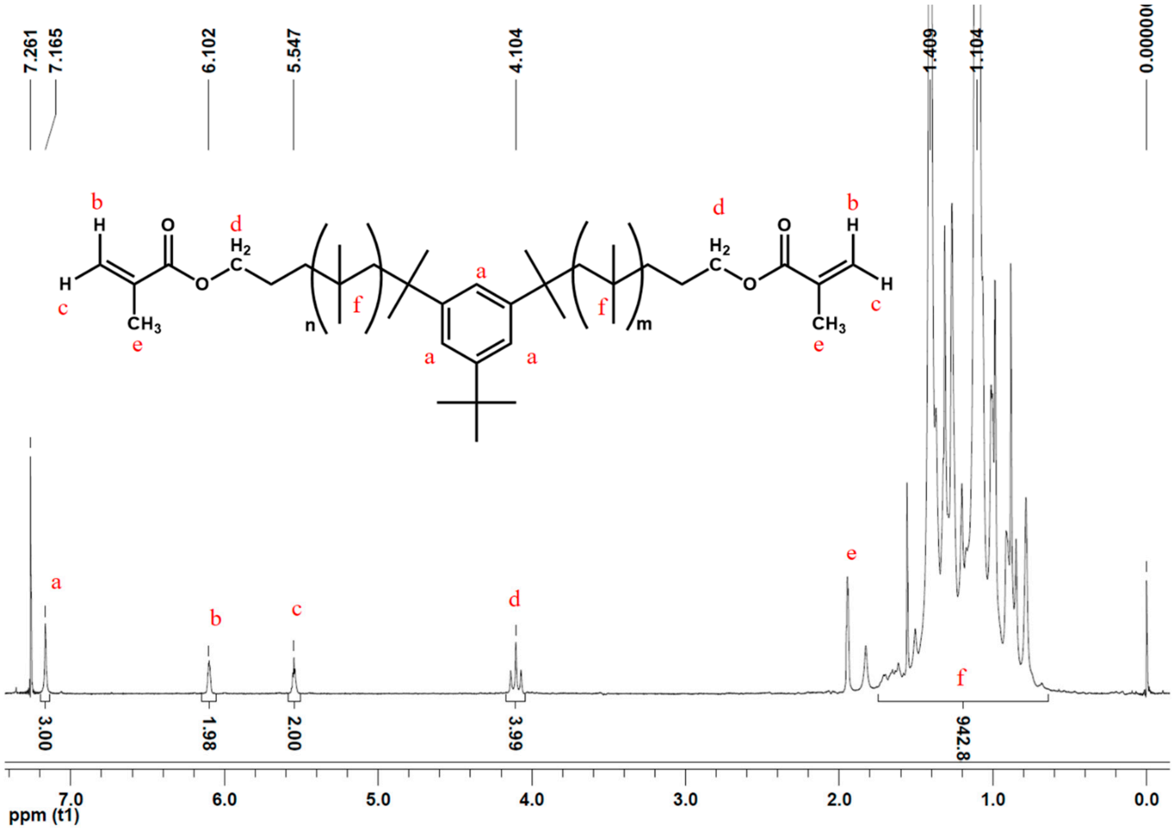

Figure S9. ^{1}H NMR spectrum of the MA–PIB–MA6.9 sample.

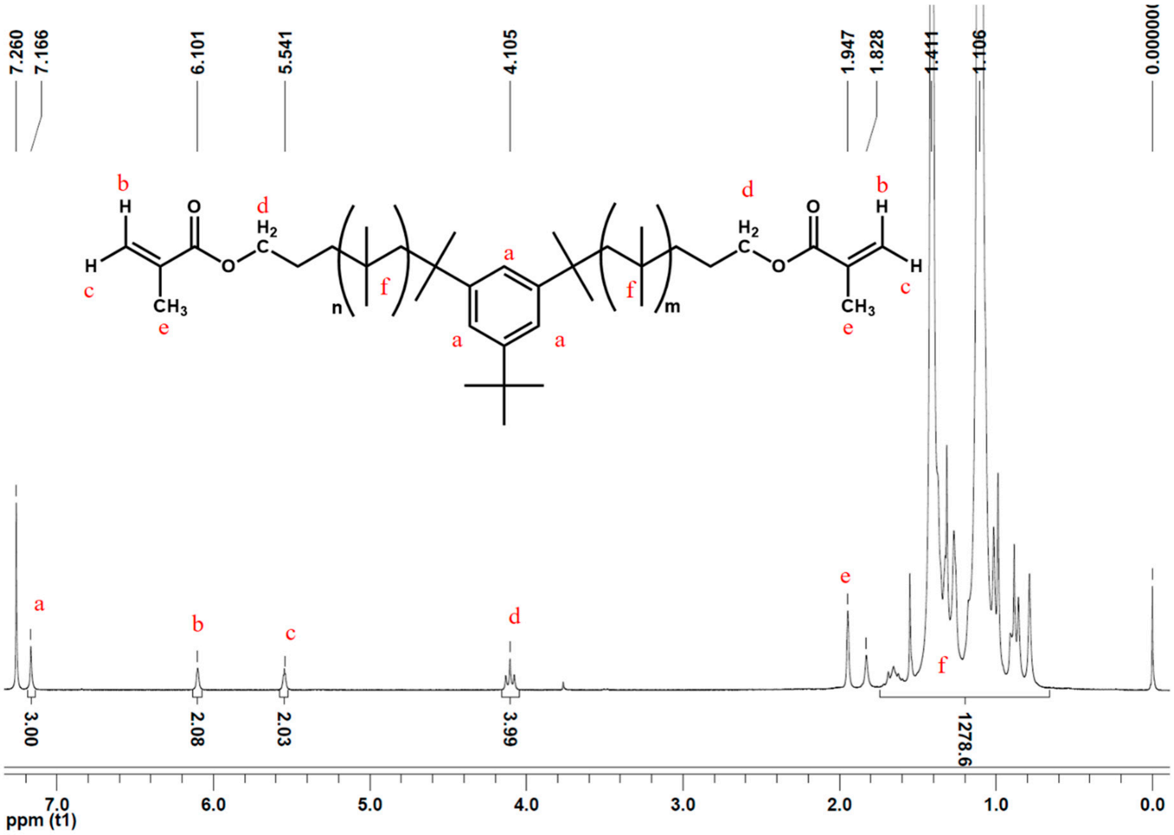

Figure S10. ^{1}H NMR spectrum of the MA–PIB–MA9.2 sample.

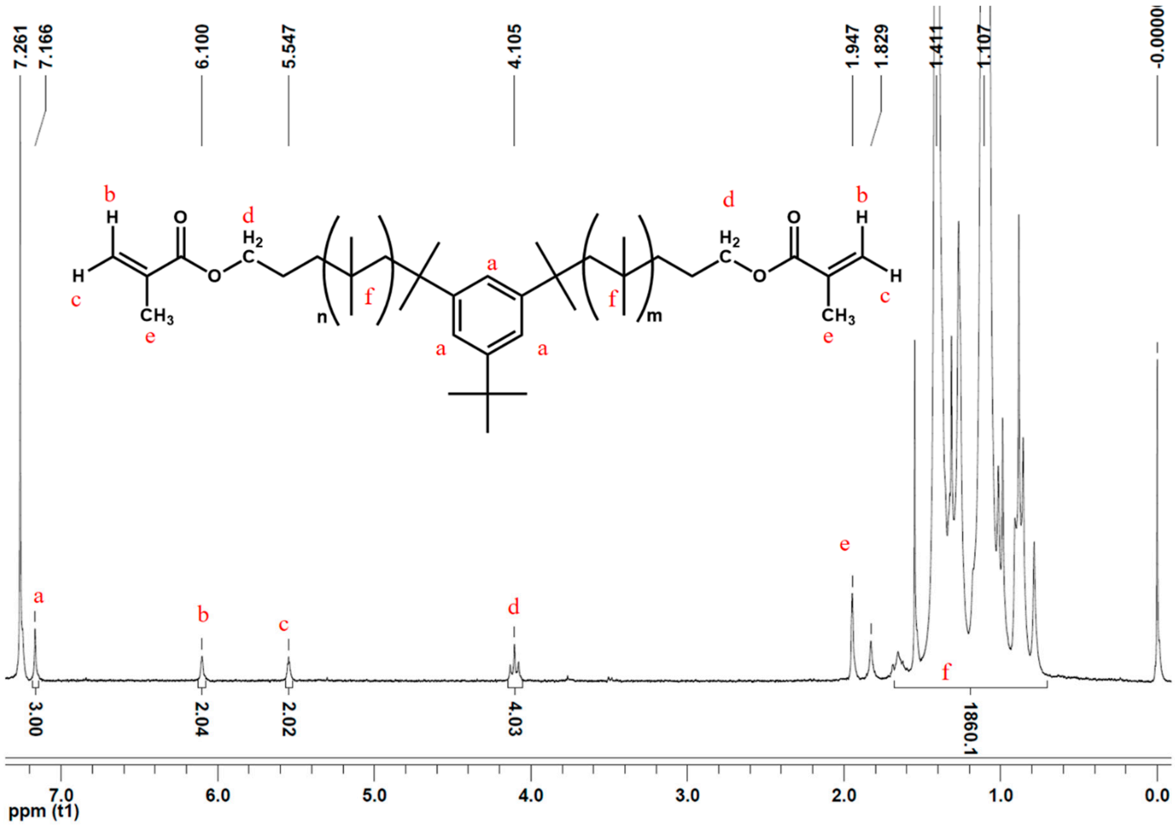

Figure S11. ^1H NMR spectrum of the MA–PIB–MA13.3 sample.
